# A Tandem Metabarcoding and Taxonomic Forensics Approach to Surveillance of Mosquito–Plant Interactions for *Culex quinquefasciatus* in Florida

**DOI:** 10.3390/insects17010013

**Published:** 2025-12-22

**Authors:** Mba-Tihssommah Mosore, Shova Mishra, Milani Villa, Bright Agbodzi, Alden S. Estep, Agne Prasauskas, Whitney A. Qualls, Daniel Killingsworth, Isik Unlu, Miranda Tressler, Rhoel R. Dinglasan, Edwin R. Burgess

**Affiliations:** 1 Entomology and Nematology Department, University of Florida, Gainesville, FL 32611, USA; 2 Department of Entomology and Plant Pathology, NC State University, Raleigh, NC 27695, USA; 3 Department of Infectious Diseases & Immunology & Emerging Pathogens Institute, College of Veterinary Medicine, University of Florida, Gainesville, FL 32610, USA; 4 USDA-ARS Center for Medical, Agricultural, and Veterinary Entomology, Gainesville, FL 32608, USA; 5 Pasco County Mosquito Control District, Odessa, FL 33556, USA; 6 Anastasia Mosquito Control District, St. Augustine, FL 32092, USA; 7 UF/IFAS Florida Medical Entomology Laboratory, Vero Beach, FL 32962, USA; 8 St. Tammany Parish Mosquito Abatement, Slidell, LA 70460, USA; 9 Volusia County Mosquito Control, New Smyrna Beach, FL 32168, USA

**Keywords:** *Culex quinquefasciatus*, nectar feeding, Nanopore sequencing, DNA metabarcoding, mosquito–plant interaction, vector control, mosquito, West Nile vector, Florida, molecular biology

## Abstract

Interactions with plants are an important aspect of mosquito vector biology and ecology because mosquitoes often use plants as nutritional resources. Plant DNA metabarcoding is a recent approach to address this topic. *Culex quinquefasciatus* is a vector of several important pathogens in Florida and is subject to significant control efforts, but no studies to date have explored what plants they may feed on. We addressed this by conducting a statewide collection of *Cx. quinquefasciatus* from six Florida counties and subjected them to plant DNA metabarcoding and cross-referenced the identified plants to a Florida state plant voucher database for each sampled county. A total of 30 families and 47 genera were identified. Plants included many that are cultivated and non-cultivated in Florida, including backyard garden produce, grasses, and some invasive species. Concurrence of plant families and genera was generally high in the respective sampled counties based on available plant vouchers. These data provide the first statewide survey of plants associated with *Cx. quinquefasciatus* in Florida. This information serves as an important basis for confirming plant taxa that are important to *Cx. quinquefasciatus*, which could translate to the development of better control strategies and tools.

## Simple Summary

Sugar is an important nutritional resource for mosquitoes that they often acquire from plants. Mosquito–plant interactions have been proposed as significant drivers of mosquito vector abundance and prevalence of mosquito-borne pathogens. A recent focus on mosquito–plant interactions has used sensitive techniques in molecular biology to screen for plant-specific genes from field-collected mosquitoes to identify plant families, genera, and species. This technique has been underutilized for mosquito species in the United States. *Culex quinquefasciatus* is a vector of many important pathogens and is found abundantly in Florida. The subject of this study was to produce a list of plants found in *Cx. quinquefasciatus* adult females using molecular detection of a plant-specific gene, *rbcL*, and to cross-reference the identified plants to a plant voucher database for the state of Florida. *Culex quinquefasciatus* were found to use a wide range of plants, including both cultivated and non-cultivated plants, and the majority of the plants had representative vouchers for their respective sampling location. Understanding plant interactions in mosquitoes could be leveraged to develop new control strategies and thus reduce the public risk of mosquito-borne pathogens.

## 1. Introduction

Like so many insects, mosquitoes have an important relationship with plants. Among the most important aspects of this relationship is the direct acquisition of plant-derived nutritional resources, such as nectar and extra-floral nectar, but also from indirect sources such as honeydew deposited by phytophagous insects [1]. While plant-derived sugars are often considered the most important factor in plant feeding by mosquitoes, other components, such as amino acids, salts, and vitamins, play important roles in their survival and normal physiological function (reviewed in [2]). Thus, to be inclusive of all the nutrients mosquitoes acquire during direct and indirect plant feeding, without implying that mosquitoes seek or utilize one nutrient more than the others, we will hereafter refer to this process as a mosquito–plant interaction.

Mosquito–plant interactions have strong implications in mosquito ecology and disease risk [3,4]. Particular interest has been paid to agricultural intensification [3], urbanization [5], and the impact of invasive plant species (reviewed in [6]), mainly in malaria-endemic countries. While their manifestations are likely different, these topics are also of relevance to the United States (U.S.). To meet the demands of food availability in the U.S., agricultural intensification will likely need to double by 2050 [7]. Agricultural intensification requires greater water inputs, which could create more larval habitat, and monoculture crops may increase the abundance of plant nutritional resources for adult mosquitoes. By 2040, an estimated 12.3 million hectares of U.S. agricultural land will be converted to urban areas [8], with predicted increases in mosquito-borne disease risk due to greater human population density and associated human practices that increase mosquito vector populations [9]. Urban greening, an effort to increase plant diversity in urban spaces to support pollinators and enhance aesthetics, risks introducing plants that are not only nutritious to pollinators but also mosquitoes [5]. That invasive plant species are considered important nutritional resources for mosquito vectors (reviewed in [6]) is especially concerning for the U.S., where some of the highest numbers of new invasive plant species have been reported over the last four decades [10]. However, a lack of basic information on mosquito–plant interactions in the U.S. currently limits exploration of these higher-order topics.

Identifying mosquito–plant interactions could also lead to new population control tools. Attractive toxic sugar baits (ATSBs) have garnered considerable attention for the control of mosquito vectors worldwide (reviewed in [11]). ATSBs were inspired by the plant feeding requirements of mosquito vectors, which utilize a volatile attractant that serves to lure adult mosquitoes and encourages feeding on an insecticide-laced liquid sugar source behind a membrane. The attractants for ATSBs usually include extracts of overripe or rotting fruits, but a better understanding of mosquito–plant interactions has been proposed as a way to improve their attractivity and ability to compete with nearby plants for mosquito visits [12].

While wide-scale surveys of mosquito–plant interactions in the U.S. are currently limited, a recent meta-analysis supports that many mosquito genera are more frequently associated with certain plant families, including Apocynaceae (e.g., dogbane, milkweed), Asteraceae (e.g., daisy, sunflower), Hypericaceae (e.g., St. John’s wort), Rosaceae (e.g., roses, many trees and bushes cultivated for fruits), and Fabaceae (e.g., legumes) [12]. Less frequently identified mosquito–plant interactions appear to be either mosquito genus-specific or a result of plant family availability in the studied regions. Some studies have also confirmed that mosquitoes have a ranked attraction to specific plant species in the field [13,14], as well as in controlled experiments with plants that are both non-cultivated [15] and cultivated [16]. Thus, mosquitoes appear to have preferences for plants and are unlikely to be generalist “scavengers” of any available plant (reviewed in [2]).

To date, mosquito–plant interactions have been resolved using several methods, including biochemical sugar quantification with anthrone [17,18] and plant-associated sugar identification with gas chromatography [1,19]. Taxonomic identification of plants fed on by mosquitoes has been done through the identification of plant secondary metabolites [20], observational studies of mosquito visitation [21], and metabarcoding molecular approaches, including first- and second-generation sequencing [13,14,22,23,24]. One benefit of utilizing second-generation sequencing is the ability to identify multiple plants in a mosquito, a limitation of first-generation sequencing (e.g., Sanger) approaches in making unambiguous taxonomic calls (e.g., [24]).

Metabarcoding is among the more recent approaches to wide-scale surveys of mosquito–plant interactions. Studies in mosquitoes have used a range of conserved chloroplast gene regions, including *matK* [14], *trnH-psbA* [13,14], *atpB* [25], and most commonly *rbcL* [13,23,24]. The *rbcL* gene (ribulose-1,5-bisphosphate carboxylase/oxygenase large subunit) can reliably identify plants to the taxonomic levels of family and genus, with significantly reduced accuracy to identify species unless additional gene regions are used to draw consensus [26,27]. However, *rbcL* is still among the best genes to target for plant metabarcoding due to the availability of large sequence databases and primer pairs that cover approximately 95% of available sequences [28]. Although many of these studies present an abundance of taxonomic information on plants that are identified in mosquito samples, most rely only on molecular identification to draw conclusions on mosquito–plant interactions and seldom include additional supporting experiments or environmental information to strengthen the associations.

Often underutilized resources for metabarcoding studies on mosquito–plant interactions are plant voucher databases, which are broadly available at the continent, country, and regional levels. With limited species-level accuracy of *rbcL* metabarcoding, plant voucher databases could help corroborate molecular identification of plant species by confirming the regional presence from which the mosquito samples were collected. One caveat is that this resource can be somewhat incomplete if vouchers do not exist for cultivated or invasive plants.

Because few studies have been conducted on mosquito–plant interactions in the U.S., the present study focused on *Culex quinquefasciatus*, an important West Nile virus vector [29,30], collected from six counties spanning the geographic latitude of the state of Florida. Additionally, this study provides sample processing protocols, bioinformatics pipelines, dataset filtering tools, and raw datasets that will enable future studies to be conducted on mosquito–plant interactions using metabarcoding, to improve mosquito control interventions, and narrow knowledge gaps on this important aspect of mosquito biology.

## 2. Materials and Methods

### 2.1. Mosquitoes and Sampling Sites

**Figure 1:**
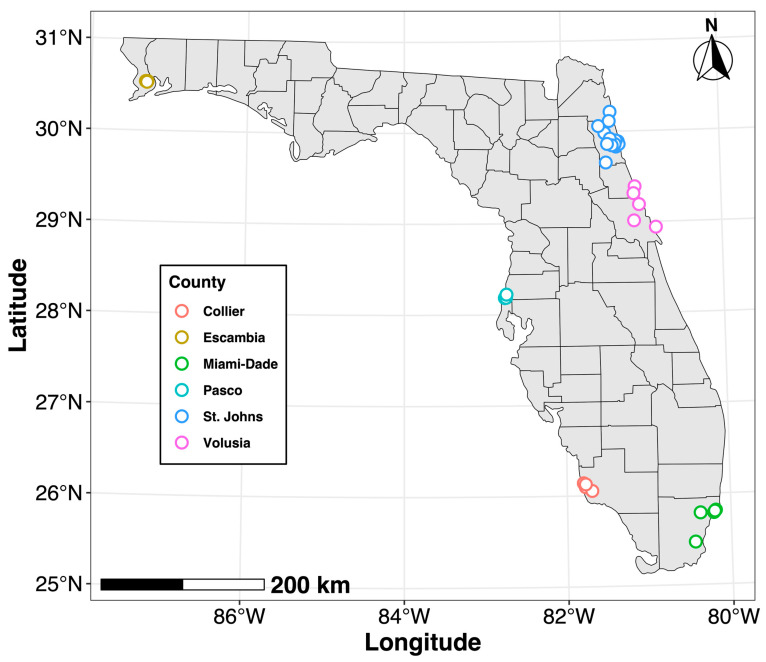
Map of field sites in Florida where adult female *Culex quinquefasciatus* were collected in April–October 2023.

A total of 1944 non-blood-engorged *Culex quinquefasciatus* adult females were collected between April and October 2023, in at least three residential areas from six mosquito control districts, each located in one of six Florida counties: Collier, Escambia, Miami-Dade, Pasco, St. Johns, and Volusia (Figure 1). Latitudes and longitudes of sample sites are not reported at the request of the mosquito control districts to maintain the anonymity of the residents at these sites. The sites were populated by typical residential plant types, including lawn, coniferous and non-coniferous trees, native plants, as well as cultivated decorative and consumable plants (i.e., fruits and vegetables) in backyard gardens. Plant species at each site were not documented during sampling. All mosquitoes were collected overnight using a variety of trap types, immediately put on dry ice, and shipped overnight to the University of Florida, where they were immediately transferred to −80 °C and stored until use.

### 2.2. External Plant Contaminant Rinse Protocol

**Figure 2:**
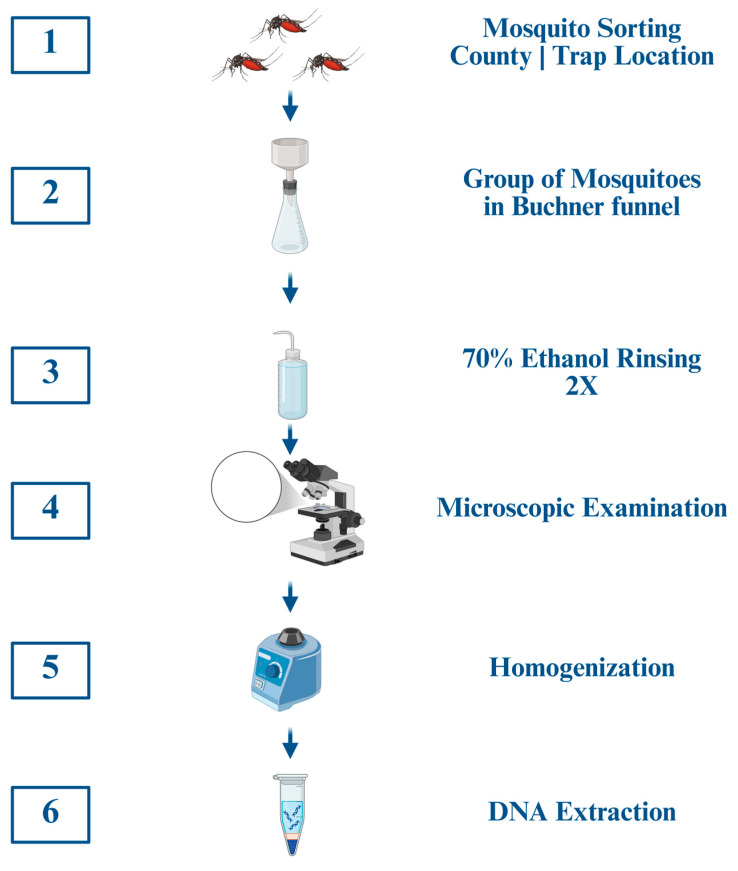
**External plant contaminant rinse protocol.** A standard six-step procedure was used to remove external contaminants from mosquitoes. Mosquito pools from all six counties were rinsed and homogenized. All steps were performed under sterile conditions. Created in BioRender. Estep, A. (2025); https://BioRender.com/1pfeimg (accessed on 25 November 2025).

**Figure 3:**
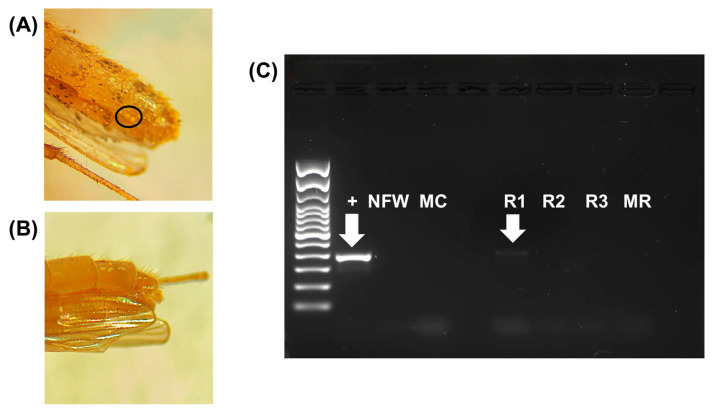
**Representative stereoscopic images of mosquitoes before and after rinsing and gel image of PCR-amplified rinsates and mosquitoes.** Adult female *Culex quinquefasciatus* were coated in pine pollen (**A**). The black circle surrounds small yellow pollen grains. Mosquitoes were then rinsed three times with 70% ethanol to remove pollen (**B**). Both pollen and some abdominal scales were rinsed off in this process. (**C**) Lanes represent *rbcL* gene amplicons of controls and rinses visualized in a 1.5% agarose gel. Lanes are as follows: + = pine pollen positive control; NFW = nuclease-free water negative control; MC = homogenized colony mosquito negative control; R1 = rinse 1; R2 = rinse 2; R3 = rinse 3; MR = homogenized, rinsed mosquito. The DNA extraction and amplification protocols are described in Section 2.3 and Section 2.4.

The trapping period fell outside of the period when pine pollen is abundant in Florida, which usually occurs from late winter through early spring. However, there was occasionally some pine pollen on the external cuticle and setae of the mosquitoes. Thus, a protocol was established to rinse off external plant contaminants from the mosquitoes (Figure 2) and is consistent with previous studies [13,14,23,24,25]. Pools of three mosquitoes (1944 total mosquitoes = 648 pools) were sorted based on county and trap location and placed in a ceramic Büchner funnel (1 mm hole size) that was sealed with a rubber stopper to a 2 L Erlenmeyer flask. The Erlenmeyer flask had an external port with a vacuum pump attached so that rinsates would be drawn into the flask from the funnel. Two successive rinses of ~20 mL 70% ethanol were done on each group of three mosquitoes, visually inspected for plant material on the outside of the mosquitoes using a stereoscope, and then the mosquitoes were transferred to 2.0 mL screw cap centrifuge tubes for homogenization and DNA extraction. Preliminary PCR studies, conducted to ensure the rinsing procedure effectively removed external contamination, amplified pine DNA in the first ethanol rinsate, with no amplification in a second or third rinsate, nor in the homogenized, rinsed mosquitoes of all tested mosquito pools (N = 10) (Figure 3).

### 2.3. Mosquito Homogenization and DNA Extraction

A volume of 200 µL of lysis buffer (100 mM Tris-HCl, 20 mM EDTA, 1.4 M NaCl, 0.2% mercaptoethanol, 2% cetrimonium bromide (CTAB), pH 8.0) was added to the tubes of rinsed mosquitoes, along with 4–5 zirconium beads (2.3 mm dia.), and homogenized three times in a Bertin PreCellys Evolution bead mill homogenizer set to a custom “skeeter” setting (4500 rpm for 5 s) (Bertin Technologies, Montigny-le-Bretonneux, France). The use of CTAB in plant DNA extractions helps to neutralize plant polyphenols, polysaccharides, and other DNases and PCR inhibitors [31], as well as the high chitin content contributed by the mosquito cuticle. The tubes were then incubated at 65 °C overnight in a water bath. Following this, 200 µL of chloroform: isoamyl alcohol (24:1) was added and gently shaken by hand for approx. 20 s. The samples were then centrifuged at room temperature for 5 min at 15,000× *g*. The upper aqueous layer (DNA layer) was carefully removed and transferred to a sterile 1.5 mL centrifuge tube. This step was repeated twice. Then, DNA purification was finished using a Qiagen DNeasy Blood and Tissue Kit according to the manufacturer’s instructions (Qiagen, Hilden, Germany), with 100 µL of DNA eluted and stored at −20 °C for less than 1 week prior to PCR.

### 2.4. Amplification of Chloroplast Gene rbcL

The extracted DNA was subjected to PCR using primers that target the ribulose-1,5-bisphosphate carboxylase/oxygenase (*rbcL*) chloroplast gene. The forward and reverse primers were forward—5′-ATGTCACCACAAACAGAGACTAAAGC-3′ [32], and reverse—5′-GTAAAATCAAGTCCACCRCG-3′ [33]. These primers were initially tested on various land plants in Florida and are among the most optimal *rbcL* primers in terms of sequence coverage, power to discriminate taxonomic information, and sensitivity to amplify template DNA [28]. Amplification reactions were as follows in 0.2 mL reaction tubes: 7.5 µL nuclease-free water, 0.5 µL each of the forward and reverse primers (10 µM each), 4 µL template DNA, and 12.5 µL Platinum Hot Start Master Mix 2x (Invitrogen, Thermo Fisher Scientific, Waltham, MA, USA). The following thermal cycler conditions were used: denaturation at 95 °C for 5 min, followed by 35 cycles of denaturation at 95 °C for 30 sec, annealing at 56 °C for 30 sec, and elongation at 72 °C for 1 min. PCR was finished with a final 10 min elongation step at 72 °C. PCR products were electrophoresed on 1.5% agarose gel in 1X TAE buffer. Tubes of PCR reactions that produced bands at an expected amplicon size of ~600–610 bp were placed in −80 °C until sequencing was performed.

### 2.5. Nanopore Sequencing and Bioinformatics

Nanopore sequencing was performed following the manufacturer-provided protocol using R10 chemistry and the Native Barcoding Kit V14 (SQK-NBD114.96; Oxford Nanopore Technologies, Oxford, UK). The protocol version used was NBA_9170_v114_revM_15Sep2022 (updated 7 March 2024). Samples were prepared for Nanopore sequencing by first combining 3 µL of PCR product, 0.25 µL of Dilute DNA Control Sample (DCS), and 9 µL of nuclease-free water in 96-well plates. Two wells contained only nuclease-free water to serve as controls to detect potential barcode mis-binning and were run on every plate. The samples were then end-prepped using the NEBNext Ultra II End Repair/dA-Tailing Module (New England BioLabs Inc., Ipswich, MA, USA) in accordance with the manufacturer’s protocol. Each sample was assigned a unique barcode from the Native Barcoding Kit (Oxford Nanopore Technologies, Oxford, UK), and all barcoded samples were then pooled, cleaned with 80% ethanol, and subjected to final ligation of sequencing adapters per the manufacturer’s protocol. The adapter-ligated, barcoded library was washed with short fragment buffer, eluted in 15 µL of elution buffer, and quantified using a Nanodrop One spectrophotometer (Thermo Fisher Scientific, Waltham, MA, USA). Eluted DNA was normalized to a concentration of 50 fmol and then mixed with sequencing buffer, loading beads, and elution buffer prior to loading onto a prepared R10 flow cell (two flow cells used in this study). Sequencing was conducted on a MinION device with MinKNOW software (version 24.02.8), with electrical signals converted to base calls by Dorado (version 7.3.11). Initial read quality control was performed by the MinKNOW software with a minimum quality score of 10. Reads meeting or exceeding this threshold were binned into barcode-specific folders as FASTQ files. Barcodes were retained in the raw reads for re-binning if necessary.

Initial barcode binning of raw reads that met the default quality control threshold was subsequently subjected to higher stringency filtering to eliminate spurious barcode mis-binning that appeared in the two control wells of our 96-well plates. The sequencing summary file for each Nanopore run was filtered using command line tools to require a barcode match of >97% and a minimum barcode alignment length of 37 bases at each end of the amplicon (Supplementary Materials File S1). A maximum of 4000 reads that met these criteria were selected for each barcode using seqtk [34]. These reads were then mapped to the reference ribulose-1,5-bisphosphate carboxylase (*rbcL*) gene from *Pinus ponderosa* (NCBI Accession: KC156882) using Minimap2 [35]. The resulting FASTA files were queried against the NCBI nt database via BLAST (version 2.16.0) to generate plant taxonomic information for each barcode. The file containing all taxonomic calls of reads for each barcode was saved as an xlsx file and filtered in R version 4.4.2 [36] using the dplyr package [37] and is available for exploration (see Data Availability Statement for data file). The filtering was done to include only plant species with a read depth of 10 or more, and the threshold for percent identity was set to 99% or greater to include only the highest accuracy reads. Two amplicon filtering lengths (450 bp and 600 bp) were considered to determine their effect on the likelihood that taxonomic identification at the genus and species level could be linked to their respective Florida counties. These two filtering lengths represent a shorter sequence length that may be more frequent in degraded samples due to digestion (i.e., 450 bp) [38] and the full-length amplicon of the targeted *rbcL* region (i.e., 600 bp). The taxonomic levels of family, genus, and species identified in samples were manually referenced to the Atlas of Florida Plants database to confirm their presence or absence by county and state [39]. All plants are reported at the family and genus level unless species-level vouchers existed in their respective sampled counties and aligned with the flowering or nutrient-availability season in which the samples were collected.

### 2.6. Statistical Analyses

To determine if amplicon filtering length could affect the frequency of plant genera and species that are found in their respective counties, a 2 × 2 Chi-square test of independence was run (“450 bp” and “600 bp” × genus or species in county “yes” or “no”) using R version 4.4.2 [36]. Statistical significance was set at α = 0.05.

## 3. Results

### 3.1. Summary of Sample Processing and Amplicon Length Filtering

A total of 648 pools of *Culex quinquefasciatus* mosquitoes were screened for plant DNA using PCR amplification of the *rbcL* chloroplast gene and Nanopore sequencing, of which 223 pools produced amplicons (34.4% positivity) and all amplicons produced plant taxonomic information. The number of pools that produced taxonomic information varied by county, with Pasco County at 100% as the highest and St. Johns County at 17% as the lowest (Table 1). Whether or not genera of identified plants were vouchered in their respective counties was statistically independent of amplicon length being filtered at 450 bp or 600 bp (χ^2^ = 0.037, df = 1, *p* = 0.848). At 450 bp, there were 56/81 instances where counties had vouchers of the identified genus, while at 600 bp, there were 39/54 instances. Similarly, whether or not species of identified plants were vouchered in their respective counties was also statistically independent of amplicon filtering length (χ^2^ = 0.257, df = 1, *p* = 0.612). At 450 bp, there were 36/121 instances where counties had vouchers of the species, while 600 bp had 28/82 instances. All counts and summary statistics were thus filtered at 450 bp hereafter to maximize the number of reads for each county. At the MinKNOW default barcode alignment length (information not publicly available), 0.02% and 0.07% of barcodes were mis-binned in flow cells one and two, respectively. Mis-binned barcodes were reduced to 0.01% and 0.02% for flow cells one and two, respectively, when barcodes were re-binned using 37 bases. However, this reduced the number of available reads to approx. 30% of the number of reads before re-binning.

### 3.2. Plant Families and Genera Identified in Adult Female Culex Quinquefasciatus from Six Counties in Florida

Representation from 30 identified plant families varied across counties (Table 2). The families Fagaceae (e.g., beeches, oaks, and chestnuts) and Pinaceae (e.g., cedars, firs, hemlocks, piñons, larches, pines, and spruces) were among the most abundant reads and the only two families found across all six counties. Fabaceae (e.g., legumes, peas, and beans) were found in five of six counties (Collier, Escambia, Miami-Dade, Pasco, and Volusia), with the highest mean reads per pooled sample in Miami-Dade. Musaceae (e.g., bananas) was also found in five of six counties (Escambia, Miami-Dade, Pasco, St. Johns, and Volusia), with the highest mean reads per pooled sample in Miami-Dade. Caprifoliaceae (e.g., honeysuckles) was found across four of six counties (Escambia, Pasco, St. Johns, and Volusia), with the highest mean reads per pooled sample in St. Johns. Other families with relatively high abundance were Acanthaceae (e.g., acanthus) in St. Johns County, Apocynaceae (e.g., dogbane) in Collier County, and Solanaceae (e.g., nightshades) in Collier County. Most of the identified families had specimen vouchers in their respective counties, except for Altingiaceae and Platanaceae, although these families are found throughout the state in other counties.

Identified plant genera were not as concordant with state specimen vouchers per county as the family level identifications were. A total of 47 plant genera were identified. The greatest concordance with county specimen vouchers by genus was found in St. Johns County, with 7 out of 8 genera found in the county (87.5%). Volusia County had the next highest concordance with 13 out of 17 genera with specimen vouchers (76.5%). Escambia County had concordance with 5 out of 7 genera vouchered in the county (71.4%). Pasco (10 out of 17 genera vouchered; 58.8%), Miami-Dade (7 out of 12 genera vouchered; 58.3%), and Collier County (15 out of the 29 genera vouchered; 51.7%) had the lowest concordance with vouchered specimens of genera in their respective counties. The genera *Aegilops*, *Carpinus*, *Daucus*, *Fragaria*, *Glycine*, *Hesperocyparis*, *Laurus*, *Lindera*, *Liquidambar*, *Lithocarpus*, *Malus*, *Platanus*, *Tabernaemontana*, and *Trigonella* did not have specimens vouchered in their respective counties. However, many of these genera are vouchered in other counties in Florida.

### 3.3. Plant Species Identified in Adult Female Culex Quinquefasciatus from Six Counties in Florida with Associated Vouchers

A total of nine plant species were identified that met the criteria of having their family, genus, and species vouchered in their respective counties, with four species in concordance with the flowering season of the species (Table 3). Five other species were either wind-pollinated or produced berries (paper mulberry) but not flowers at the time of collection. *Russelia equisetiformis* and *Stenotaphrum secundatum* were each found in two separate counties. Poaceae was the most frequent family among the species.

## 4. Discussion

The present study highlights a diversity of plant taxa found in samples of *Cx. quinquefasciatus* across the state of Florida, with 47 plant genera represented across 30 families. A total of 28 of the 30 families were angiosperms, and 20 of the 30 are largely insect-pollinated (i.e., entomophilous). Of the 30 angiosperm families, eight are primarily considered wind-pollinated. Interestingly, two gymnosperm families were also identified, Cupressaceae and Pinaceae. A recent meta-analysis on mosquito–plant interactions revealed a wide range of plant families among numerous vector-relevant mosquito genera, including *Aedes*, *Anopheles*, *Culex*, *Culiseta*, *Coquillettidia*, and *Psorophora* [12]. The most frequently reported plant interactions in mosquitoes were the families Apocynaceae (e.g., dogbane, milkweed), Asteraceae (e.g., daisy, sunflower), Hypericaceae (e.g., St. John’s wort), Rosaceae (e.g., roses, many trees and bushes cultivated for fruits), and Fabaceae (e.g., legumes), with Asteraceae being the most commonly reported for *Culex* spp. It is interesting that no Asteraceae was detected in any of our samples. At the time the present study was conducted, there were 440 vouchered species in the family Asteraceae in Florida, with some representation in all 67 counties [39]. On the other hand, both Rosaceae and Fabaceae were found in the present study, with the greatest number of identified genera of all families in Fabaceae. Few of the studies referenced in [12] present data on the *Culex pipiens* complex, to which *Cx. quinquefasciatus* belongs. Narrowing their list of citations to only include the *Cx. pipiens* complex interactions with plants through either molecular surveillance or visual observations in the field narrows the list of plant families (genera, common name) to Asteraceae (*Achillea*, yarrow), Cornaceae (*Cornus*, silky dogwood) in Wisconsin, U.S. [40], Asteraceae (*Tanacetum*, tansy flower) in Sweden [41], and Asteraceae (*Tanacetum*, tansy; *Achillea*, yarrow) in Canada [42]. We note that all three of these locations are in more northern latitudes than Florida. Thus, there could be geographical or ecologically relevant factors that shift plant interactions among different mosquito taxa [12]. A previous study found many similar plant families in *Cx. quinquefasciatus* samples collected in Israel [25]. These families, identified with *rbcL* metabarcoding, included Apiaceae, Apocynaceae, Brassicaceae, Cucurbitaceae, Cupressaceae, Juglandaceae, Moraceae, Pinaceae, Poaceae, Rosaceae, and Solanceae. The study used a second metabarcoding gene, ATP synthase beta subunit (*atpB*), and found plant genera concurrent with the present study, including *Cucumis*, *Fraxinus*, *Medicago*, *Musa*, *Pinus*, *Quercus*, and *Solanum*. Although metabarcoding studies of plant interactions in *Cx. quinquefasciatus* are scant, the present study serves both the purpose of building consensus of previously documented taxa and introducing new taxa to the list of plants *Cx. quinquefasciatus* may utilize for nutritional resources.

Another important observation in the present study was the association of *Cx. quinquefasciatus* with some invasive angiosperm species. Notably, we found *Broussonetia papyrifera* (paper mulberry) and *Dioscorea* spp. in our pools. Paper mulberry is considered an abundant, invasive species in Florida, while three of five vouchered *Dioscorea* spp. are also considered abundant and invasive [39]. Similar interactions with invasive species have also been observed in *Anopheles gambiae* across Africa [22,43]. Invasive plants have been proposed as a possible challenge to managing mosquito-borne diseases because invasive plants can rapidly expand their geographic ranges and provide harborage and nutrients for mosquito vectors [6,44].

The most notable non-entomophilous angiosperms associated with *Cx. quinquefasciatus* in our samples were grasses (family Poaceae). *Zea* spp. (i.e., corn), a common backyard plant grown for consumption in Florida, is the most logical association to make with *Cx. quinquefasciatus* due to its sugar-rich tissues and availability. *Zea* spp. have also been identified in *Aedes aegypti* in Kenya [24]. Whether *Zea* spp. are attractive to mosquitoes for the sole purpose of acquiring nutrition is currently unknown. Without the availability of nectar or extrafloral nectaries, mosquitoes may acquire nutritional resources from guttation droplets that many plants produce, including grasses. Guttation is a process of water pressure regulation that pushes xylem and phloem fluids rich in proteins, minerals, and sugars through hydathode pores along the margins of leaves, mainly at night [45]. Insects utilize guttation droplets as a nutrient source [46]. High concentrations of glucose, galactose, and fructose have been found in guttation droplets from rye, wheat (i.e., *Triticum* spp.), and barley grasses [47]. Even simple observational studies on mosquito interactions with guttation droplets are absent from the literature and would be a meaningful topic to explore in future studies.

Two other plant families identified in the present study, with the most abundant reads of all plant families, were Fagaceae and Pinaceae, which have also been found in previous metabarcoding studies with *Cx. quinquefasciatus* [25]. Fagaceae (i.e., oaks) do not produce nectaries and are not considered entomophilous plants. However, oaks in Florida are commonly afflicted with heavy aphid infestations, including introduced species such as the Southern oak thelaxid, *Thelaxes suberi* [48] and the woolly oak aphid, *Stegophylla brevirostris* [49]. Aphids produce honeydew, an excreted sugary waste from the plants they feed on. Mosquitoes are known to utilize honeydew as a nutritional resource [2,50]. Pinaceae (i.e., pines) also does not produce nectaries and is not considered entomophilous. Further, the sampling period in this study did not occur when pine pollen is most abundant in the environment, making pine pollen an unlikely contaminant. However, there is also some evidence that mosquitoes utilize honeydew produced by aphids infesting Pinaceae. An earlier study collected mosquitoes in sand pines infested with aphids, but no mosquitoes were observed in nearby sand pines that did not have aphids [51]. Honeydew may also be an advantageous nutritional resource to primarily nocturnal host-seeking mosquito species like *Cx. quinquefasciatus* [52], because honeydew will be present during host-seeking hours while some nyctinastic (i.e., nocturnally closing) flowering plant species are unavailable. Adult male *Aedes sollicitans* have been observed feeding on aphid honeydew deposited on Spanish needle during crepuscular-nocturnal hours [50]. Some evidence exists that *Cx. pipiens* complex mosquitoes visit floral sugar sources at night [53]. A meaningful add-on to future metabarcoding studies of mosquito–plant interactions would be to include surveillance of aphid or other hemipteran DNA signatures, which are possible to detect in honeydew samples [54].

The present study has expanded on the typical metabarcoding approach to mosquito–plant interactions by also inspecting a state-curated plant specimen voucher system, to confirm whether plants identified in *Cx. quinquefasciatus* samples would be likely found in their respective counties. The voucher system utilized includes primarily non-cultivated native and non-native plants. Many plant families found in the present study are cultivated or used in landscaping throughout Florida, including Platanaceae (sycamore trees), Altingiaceae (sweetgum), and Betulaceae (birch, hornbeam), among others. Thus, it is entirely possible that plants without county vouchers could still likely be found in their respective counties. In lieu of a voucher system, taxonomic information of plants detected in mosquito samples could be bolstered with a second barcoding primer set utilizing a different gene, such as *matK*, *trnH-psbA*, or *atpB,* to achieve a species-level identification that *rbcL* alone is usually not capable of.

Another point worthy of discussion is the validity that plants identified in mosquito samples were likely fed on and were not merely external contamination of the mosquito samples. While this can never be completely resolved using only molecular metabarcoding, sample processing strategies can minimize the effect of external contamination. Gymnosperms and wind-pollinated angiosperms generally do not have the “sticky” coating, called pollenkitt, commonly found on entomophilous plant taxa pollen that enables it to stick to pollinators [55]. However, pollenkitt is soluble in a wide range of solvents [56], which enables effective removal of this type of contamination, as was demonstrated in our methods. Further, the mosquito proboscis inner diameter has been reported in some species to be around 20–30 μm, depending on sex [57,58]. Entomophilous plants often have pollen diameters exceeding the size of the proboscis inner diameter [59], making it unlikely but not impossible that nectar contaminated with pollen would influence our results. However, this is not universal across all flowering plants, as the smallest land plant pollen, from *Myosotis* spp., has been measured at approx. 3.2 μm width [60]. Pollen from the gymnosperm family Pinaceae has relatively large pollen grains, exceeding 60 μm in some species [61]. As with any environmental sampling approach, including molecular metabarcoding of mosquito–plant interactions, contamination can never be completely ruled out. Thus, we urge readers to view these types of studies as a starting point to narrow down the list of target plants for further inquiry.

With plant-derived nutritional resources being a common part of mosquito vector biology, there are numerous ways the information can be used from the present study and those like it. For example, using preferred plants as baiting components in ATSB traps has been proposed for the *Culex pipiens* complex, with some demonstrated efficacy to attract [15]. Mosquito–plant interaction studies like the present one could also inform the discovery of advantageous visual, olfactory, and gustatory cues that make plants attractive to mosquitoes, leading to improved ATSP traps or other technologies that exploit mosquito plant feeding [62]. Altering plant assemblages in the environment could also lead to sustainable, non-chemical control of vectors by reducing plants they often feed on or by reducing the presence of animal hosts. This has been demonstrated with *Culex pipiens* and *Culex restuans* with the removal of invasive honeysuckle [63]. The present study concludes that Florida *Cx. quinquefasciatus* likely utilize a wide range of plant families and genera as nutritional resources, but with some clear preferences for certain families and genera. Adding this data to the general body of knowledge helps to build consensus around what types of plants mosquitoes use as nutritional resources. A cornerstone of vector control is vector ecology. Knowing where and when mosquitoes may be found based on the types of plants they use could improve control strategies and lead to the development of better control tools.

## Figures and Tables

**Table 1:** Total pools of adult female *Culex quinquefasciatus* tested, total pools that produced positive hits (i.e., amplicons and plant taxonomic information) in six Florida counties.

County	Total Pools	Total Positive	Percent Positive
Collier	155	87	56.1%
Escambia	55	10	18.2%
Miami-Dade	204	35	17.2%
Pasco	29	29	100%
St. Johns	100	17	17.0%
Volusia	105	45	42.9%

**Table 2:** Mean reads of plant families per number of pooled adult female *Culex quinquefasciatus* samples in six Florida counties. Numbers in bold have vouchered specimens of the family or genus in the respective county.

	County					
	Collier	Escambia	Miami-Dade	Pasco	St. Johns	Volusia
Family *Genus*	Mean Reads per Pooled Mosquito Samples (Number of Pools) ^a^					
Acanthaceae	-	-	-	-	**330.0 (2)**	-
*Avicennia*	-	-	-	-	**330.0 (2)**	-
Altingiaceae	-	-	14.0 (1)	-	-	-
*Liquidambar*	-	-	14.0 (1)	-	-	-
Apiaceae	**10.5 (2)**	-	-	-	-	-
*Daucus*	10.5 (2)	-	-	-	-	-
Apocynaceae	**119.3 (12)**	-	-	**28.0 (1)**	**51.0 (1)**	**6.5 (2)**
*Tabernaemontana*	119.3 (12)	-	-	28.0 (1)	-	6.5 (2)
*Orthosia*	-	-	-	-	**51.0 (1)**	-
Arecaceae	**23.0 (1)**	-	-	-	-	-
*Sabal*	**23.0 (1)**	-	-	-	-	-
Betulaceae	201.0 (1)	-	-	-	-	-
*Carpinus*	201.0 (1)	-	-	-	-	-
Brassicaceae	**26.0 (2)**	-	-	-	-	-
*Brassica*	**26.0 (2)**	-	-	-	-	-
Cannabaceae	**96.0 (2)**	-	-	**82.0 (1)**	-	-
*Celtis*	**96.0 (2)**	-	-	**82.0 (1)**	-	-
Caprifoliaceae	-	**224.0 (4)**	-	**209.1 (11)**	**965.0 (1)**	**13.8 (5)**
*Lonicera*	-	**224.0 (4)**	-	**209.1 (11)**	**965.0 (1)**	**13.8 (5)**
Cucurbitaceae	**9.0 (1)**	-	-	**9.0 (3)**	-	-
*Cucumis*	**9.0 (1)**	-	-	**9.0 (3)**	-	-
Cupressaceae	**12.4 (15)**	-	-	-	-	**21.2 (5)**
*Hesperocyparis*	6.7 (3)	-	-	-	-	13.7 (3)
*Juniperus*	**2.8 (4)**	-	-	-	-	**7.5 (2)**
*Taxodium*	**3.0 (8)**	-	-	-	-	-
Dioscoreaceae	**3.7 (3)**	-	**18.0 (1)**	-	**13.0 (2)**	-
*Dioscorea*	**3.7 (3)**	-	**18.0 (1)**	-	**13.0 (2)**	-
Fabaceae	**121.8 (9)**	**5.0 (2)**	**2492.0 (3)**	**58.5 (2)**	-	**56.6 (10)**
*Acacia*	**18.0 (1)**	-	-	-	-	-
*Arachis*	5.3 (4)	-	-	-	-	**56.6 (10)**
*Desmodium*	**75.5 (2)**	-	-	-	-	-
*Medicago*	11.0 (1)	-	**58.0 (1)**	-	-	-
*Trigonella*	12.0 (1)	-	-	-	-	-
*Glycine*	-	5.0 (2)	-	**58.5 (2)**	-	-
*Cassia*	-	-	**2420.0 (1)**	-	-	-
*Cenostigma*	-	-	14.0 (1)	-	-	-
Fagaceae	**888.0 (250)**	**446.8 (5)**	**525.5 (48)**	**141.8 (30)**	**306.2 (12)**	**683.0 (116)**
*Lithocarpus*	3.2 (45)	-	-	-	-	6.6 (32)
*Quercus*	**884.8 (205)**	**446.8 (5)**	**525.5 (48)**	**141.8 (30)**	**306.2 (12)**	**676.4 (84)**
Juglandaceae	-	-	**23.0 (2)**	**77.5 (2)**	-	-
*Juglans*	-	-	23.0 (2)	77.5 (2)	-	-
Lauraceae	**9.6 (10)**	-	-	-	-	-
*Laurus*	6.4 (5)	-	-	-	-	-
*Lindera*	3.2 (5)	-	-	-	-	-
Moraceae	-	-	-	-	-	**23.0 (1)**
*Broussonetia*	-	-	-	-	-	**23.0 (1)**
Musaceae	-	18.0 (1)	**272.0 (1)**	**27.0 (3)**	38.0 (1)	**41.0 (2)**
*Musa*	-	18.0 (1)	**272.0 (1)**	**27.0 (3)**	38.0 (1)	**41.0 (2)**
Nyssaceae	100.6 (16)	-	33.5 (2)	**31.0 (1)**	-	**8.5 (2)**
*Nyssa*	100.6 (16)	-	33.5 (2)	**31.0 (1)**	-	**8.5 (2)**
Oleaceae	-	-	-	-	-	**13.0 (1)**
*Fraxinus*	-	-	-	-	-	**13.0 (1)**
Pinaceae	**1256.4 (159)**	**62.5 (4)**	**296.9 (48)**	**203.0 (38)**	**19.5 (8)**	**206.3 (51)**
*Pinus*	**1256.4 (159)**	**62.5 (4)**	**296.9 (48)**	**203.0 (38)**	**19.5 (8)**	**206.3 (51)**
Plantaginaceae	**26.0 (1)**	-	**35.3 (3)**	-	-	**8.3 (3)**
*Russelia*	**26.0 (1)**	-	**35.3 (3)**	-	-	8.3 (3)
Platanaceae	54.0 (1)	-	53.0 (1)	-	-	-
*Platanus*	54.0 (1)	-	53.0 (1)	-	-	-
Poaceae	**49.0 (1)**	**176.7 (4)**	-	**193.0 (6)**	-	**57.9 (18)**
*Zea*	49.0 (1)	**20.7 (3)**	-	43.0 (2)	-	-
*Triticum*	-	**156.0 (1)**	-	70.0 (1)	-	-
*Aegilops*	-	-	-	**12.0 (1)**	-	-
*Paspalidium*	-	-	-	**11.0 (1)**	-	**6.8 (9)**
*Stenotaphrum*	-	-	-	**57.0 (1)**	-	**51.1 (9)**
Rhizophoraceae	**13.0 (1)**	-	-	-	-	-
*Rhizophora*	**13.0 (1)**	-	-	-	-	-
Rosaceae	**32.0 (2)**	-	-	**13.0 (2)**	-	-
*Malus*	32.0 (2)	-	-	-	-	-
*Fragaria*	-	-	-	13.0 (2)	-	-
Solanaceae	**724.8 (5)**	-	-	-	-	**7.0 (2)**
*Solanum*	**724.8 (5)**	-	-	-	-	**7.0 (2)**
Ulmaceae	-	-	-	-	**27.0 (1)**	**24.0 (1)**
*Ulmus*	-	-	-	-	**27.0 (1)**	**24.0 (1)**
Verbenaceae	**16.0 (1)**	-	-	-	-	-
*Phyla*	**16.0 (1)**	-	-	-	-	-
Vitaceae	-	-	-	**63.0 (1)**	-	-
*Vitis*	-	-	-	**63.0 (1)**	-	-
**Positive pools**	**87**	**10**	**35**	**29**	**17**	**45**

^a^ Mean reads per sample are unique plant species identified within each family or genus divided by the total number of sample pools in which they were found. Number of pools can exceed the total positive pools for each county because pools often had the multiple plant species identified within each plant family or genus. Example: Collier County had *Quercus robur* (Fagaceae) at 15,751 reads across 48 positive pools and *Quercus virginiana* (Fagaceae) at 23,517 reads across 50 positive pools. This would be presented as 23,517 + 15,751 (39,268) reads divided by 48 + 50 (98) pools, or 400.7 mean reads of Fagaceae or *Quercus* per pool. Please see full dataset under Data Availability Statement. Bold font represents families and genera that have confirmed vouchers in their respective counties.

**Table 3:** Identified plant species from adult *Culex quinquefasciatus* in six Florida counties. All plant species listed have vouchers in the associated counties and only one species within their genus is vouchered in Florida.

County	Family	Scientific Name	Common Name	Flowering at Time of Collection (Season)
Collier	Plantaginaceae	*Russelia equisetiformis*	Firecracker plant	Yes (fall)
Escambia	Poaceae	*Triticum aestivum*	Wheat	NA ^a^ (fall)
Escambia	Poaceae	*Zea mays*	Corn	NA ^a^ (fall)
Miami-Dade	Fabaceae	*Cassia fistula*	Golden shower tree	Yes (spring)
Miami-Dade	Plantaginaceae	*Russelia equisetiformis*	Firecracker plant	Yes (spring)
Pasco	Poaceae	*Stenotaphrum secundatum*	St. Augustine grass	NA ^a^ (summer)
St. Johns	Apocynaceae	*Orthosia scoparia*	Leafless swallowwort	Yes (summer)
Volusia	Moraceae	*Broussonetia papyrifera*	Paper mulberry	No ^b^ (summer)
Volusia	Poaceae	*Stenotaphrum secundatum*	St. Augustine grass	NA ^a^ (summer)

^a^ NA = Species are wind-pollinated and do not produce externally available sugar resources such as nectar. ^b^ Paper mulberry flowers in late spring but bears fruit throughout summer, when the samples were collected.

## Data Availability

Raw data outputs are available in NCBI BioProject: PRJNA1359414. Filtered data used to generate Table 1 is available in repository at: https://ufdc.ufl.edu/ir00012354/00001/downloads (accessed on 18 December 2025).

## Supplementary Materials

The following supporting information can be downloaded at: https://www.mdpi.com/article/10.3390/insects17010013/s1, Supplementary File S1 Bioinformatics Pipeline.

## Abbreviations

*Cx*	Culex
ATSB	Attractive toxic sugar bait
CDC	Center of Disease Control, United States
*rbcL*	ribulose-1,5-bisphosphate carboxylase/oxygenase large subunit gene
*matK*	megakaryocyte-associated tyrosine kinase gene
*trnH-psbA*	interspacer region of the transfer RNA (ribonucleic acid) for histidine gene and the D1 protein in photosystem II gene.
*atpB*	beta-subunit of ATP (adenosine triphosphate) synthase gene
U.S.	United States of America
DNA	deoxyribonucleic acid
PCR	polymerase chain reaction
CTAB	cetrimonium bromide
NCBI	National Center for Biotechnology Information
BLAST	Basic Local Alignment Search Tool
